# Analyses of Old “Prokaryotic” Proteins Indicate Functional Diversification in *Arabidopsis* and *Oryza sativa*

**DOI:** 10.3389/fpls.2016.00304

**Published:** 2016-03-15

**Authors:** Anupama Singh, Minesh Jethva, Sneh L. Singla-Pareek, Ashwani Pareek, Hemant R. Kushwaha

**Affiliations:** ^1^School of Computational and Integrative Sciences, Jawaharlal Nehru UniversityNew Delhi, India; ^2^International Center for Genetic Engineering and BiotechnologyNew Delhi, India; ^3^Plant Stress Biology, International Center for Genetic Engineering and BiotechnologyNew Delhi, India; ^4^Stress Physiology and Molecular Biology Laboratory, School of Life Sciences, Jawaharlal Nehru UniversityNew Delhi, India

**Keywords:** *Oryza sativa*, *Arabidopsis*, old “prokaryotic” proteins, evolution, domain

## Abstract

During evolution, various processes such as duplication, divergence, recombination, and many other events leads to the evolution of new genes with novel functions. These evolutionary events, thus significantly impact the evolution of cellular, physiological, morphological, and other phenotypic trait of organisms. While evolving, eukaryotes have acquired large number of genes from the earlier prokaryotes. This work is focused upon identification of old “prokaryotic” proteins in *Arabidopsis* and *Oryza sativa* genome, further highlighting their possible role(s) in the two genomes. Our results suggest that with respect to their genome size, the fraction of old “prokaryotic” proteins is higher in *Arabidopsis* than in *Oryza sativa*. The large fractions of such proteins encoding genes were found to be localized in various endo-symbiotic organelles. The domain architecture of the old “prokaryotic” proteins revealed similar distribution in both *Arabidopsis* and *Oryza sativa* genomes showing their conserved evolution. In *Oryza sativa*, the old “prokaryotic” proteins were more involved in developmental processes, might be due to constant man-made selection pressure for better agronomic traits/productivity. While in *Arabidopsis*, these proteins were involved in metabolic functions. Overall, the analysis indicates the distinct pattern of evolution of old “prokaryotic” proteins in *Arabidopsis* and *Oryza sativa*.

## Introduction

Evolution is a process that results in change in the frequency of alleles within a gene pool, across generations. It enables species to cope up with various environmental conditions, both abiotic and biotic (Helena and Sue Barnes, 1989). These phenotypic variations are the result of genome evolution which takes place through various processes such as mutations, transfer of genes and genomes between species, amplification and mobility of DNA, and amplification and homogenization of tandemly repeated DNA sequences. These processes also lead to the subtle modifications in the pre-existing genes that lead to the evolution of new genes with novel functions, which further results in lineage-or species-specific phenotypic traits in an organism (Kaessmann, 2010). Several mechanisms have been theorized to explain appearance of new genes with novel functions in various organisms. These mechanisms involve duplication, lateral transfer, horizontal transfer, fusion and fission, and *de novo* genesis of genes whereas other mechanisms such as non-disjunction, tandem duplication, retropositions, and transpositions assist the gene duplication events (Ohno, 1970; Jiang et al., 2004; Morgante et al., 2005). In addition, gene duplication events also occur through rearrangements and subsequent repair of staggered breaks (Ranz et al., 2007). Genes which come from the unrelated genomes are considered as probable case of lateral gene transfer. This is the most common mechanism for gene induction in prokaryotes, and has also been reported in genomes of cellular organelles such as mitochondria, chloroplasts, and nucleus in eukaryotes (Roger, 1999). The evolution in eukaryotes has been hypothesized as an outcome of the massive influx of the bacterial genes through primary and secondary endo-symbiosis and horizontal transfer of genes. The well-known evidence of such phenomenon is the existence of mitochondria and chloroplasts in plants (Timmis et al., 2004; Embley and Martin, 2006). It has been observed that non-coding regions of DNA also add new genes to the genome, a process termed as *de novo* genesis. The evolution process in the eukaryotes also occurs via loss of genes and appearance of new genes leading to the evolution of new proteins (Koonin et al., 2004; Miller and Ball, 2008).

A eukaryotic genome is comprised of heterogeneous set of genes, which apart from differing in terms of function, also have distinct evolutionary histories (Vishnoi et al., 2010). These varied evolutionary history of various genes, allow the identification of the orthologs across diverse range of species spanning vast evolutionary distances. Thus, these genes can be categorized as the “old,” depending upon the identification of their respective orthologs in wide range of species (Wolf et al., 2009; Vishnoi et al., 2010). The genes with no visible orthologs, which might have evolved due to duplication and further got drifted from the ancestral copy due to accelerated substitutions or any other evolutionary event, may be categorized as “new” genes (Ohno, 1970; Long, 2001; Lynch and Katju, 2004; Toll-Riera et al., 2009). The classified new proteins in an organism could be the result of refashioned old genes duplicated during the evolutionary process (Ohno, 1970). As proteins often evolve within the constraints of their conserved function (Ingram, 1961) and considering that the protein sequences preserve the information throughout the evolutionary process, protein sequence comparison can be considered as a powerful tool for understanding genome evolution. For example, as earlier reported, CBS (Cystathionine β-synthase) domain containing protein (CDCP) encoding genes have been hypothesized to have evolved and assumed diverse functions in plants (Kushwaha et al., 2009).

The availability of sequenced genomes for model crop plants such as *Arabidopsis* (Arabidopsis Genome Initiative, 2000) and *Oryza sativa* (Goff et al., 2002) have enabled comparative studies of these genomes. This comparative analysis is also strengthened by the fact that the two species shared a common ancestor ~150–200 million years ago (Jackson et al., 2006). Earlier, a whole genome comparative study in *Arabidopsis* and *Oryza sativa* has extended the knowledge of various genes and gene families which play important role in various abiotic stress responses (Nelson et al., 2004; Pareek et al., 2006; Yuan et al., 2007; Kushwaha et al., 2009; Mustafiz et al., 2011; Tripathi et al., 2015). Recently, one of the two component system (TCS) family member has been shown to play major role in circadian rhythm apart from its usual function in stress response signaling system (Singh A. et al., 2015). Comparative analysis of *Arabidopsis* and *Oryza sativa* genomes has been considered very useful in understanding the genomic similarities/differences across monocot/dicot divide (Liu et al., 2001; Louis, 2007). Also, previous studies have established the collinearity between *Arabidopsis* and *Oryza sativa* genomes at both genetic and physical map levels (Dodeweerd et al., 1999). Therefore, with the comparative genomics approach, genome scale differences can be identified between organisms which, in turn, can provide insights into the evolution of these organisms. The present work is focused on the identification and classification of old and new genes in *Arabidopsis* and *Oryza sativa*, along with the detailed analysis of old “prokaryotic” gene in both the genomes. The comparative analysis of the distribution of old genes presented in the paper will assist in identification of complex changes which may have accrued during evolution.

## Materials and methods

### Data for analysis

The genome sequence for *Oryza sativa* was obtained from *Oryza sativa* genome annotation project (http://rice.plantbiology.msu.edu/; Ouyang et al., 2007). All the analyses in *Oryza sativa* were performed on version 7.0 of the *Oryza sativa* genome annotation data. The genome sequence for *Arabidopsis* was obtained from the *Arabidopsis* information source TAIR (http://www.arabidopsis.org; Lamesch et al., 2012). All the analyses were performed on TAIR10 version of the resource. The prokaryotic proteins were obtained from COGs database (Phylogenetic classification of proteins encoded in complete genomes; http://www.ncbi.nlm.nih.gov/COG/) at NCBI as a complete unicellular cluster. Further, to validate the results, the orthologs were also searched using 2766 bacterial species (ftp://ftp.ncbi.nlm.nih.gov/genomes/refseq/bacteria/) available at NCBI database.

### Identification of the old “prokaryotic” proteins

The analyses were performed using the protein sequences obtained from the *Arabidopsis* and *Oryza sativa* genome database. In order to classify proteins as old and new, we first identified protein homologs in 2766 bacterial species using reciprocal BLASTP searches (*E*-value threshold 1 × 10^−6^; Tatusov et al., 1997). Proteins with an ortholog in bacteria (ftp://ftp.ncbi.nlm.nih.gov/genomes/refseq/bacteria/) were considered as old while the proteins without orthologs were considered new. These orthologs have been named as the old “prokaryotic” proteins in *Arabidopsis* and *Oryza sativa*. This approach has been earlier used to classify the genes as old or young (Wolf et al., 2009; Vishnoi et al., 2010). Further, in order to validate the results, we performed the BLASTP homolog search of the *Arabidopsis* and *Oryza sativa* protein sequences with the prokaryotic proteins obtained from COGs database.

### Domain prediction and construction of domain architectures

In order to identify domains, families, motifs, and repeats in the protein sequences identified as “old” prokaryotic proteins in both *Arabidopsis* and *Oryza sativa* using BLASTP searches, Pfam database was scanned using PfamScan program (Bateman et al., 2004). The Pfam predictions were performed locally, in order to predict the domains. For this purpose, the NCBI-BLAST (Altschul et al., 1997), HMMER (Eddy, 1998), and PfamScan (Bateman et al., 2004) programs were installed locally. The program PfamScan, systematically executes BLAST and HMMER programs to search the domain profiles from Pfam database. The entire domain profile was predicted by using the default parameters. The output of the PfamScan program was further parsed and domain architecture of various proteins was constructed using the PERL programming language. The common domains, families, motifs and repeats present in *Arabidopsis* and *Oryza sativa* were identified using the PfamScan results.

### GO analysis

In *Oryza sativa*, the GO terms were identified using the GOSlim assignment provided on *Oryza sativa* genome annotation project website (http://rice.plantbiology.msu.edu/annotation_pseudo_goslim.shtml). For *Arabidopsis*, we relied on the GOSlim assignments provided by *Arabidopsis* information source TAIR (http://www.arabidopsis.org). All the proteins identified in *Arabidopsis* and *Oryza sativa* were categorized in three broad GOSlim categories such as molecular function, biological process and cellular component. The GOSlim assignments were made for the old “prokaryotic” proteins with a corrected *p* ≤ 0.05.

### Statistical analysis

The cumulative density function (cdf) plot of the Pfam-predicted domains was prepared using MATLAB 2011a software. The two-sample Kolmogorov-Smirnov test was applied in order to compare the distribution of the domain architecture pattern of proteins in both *Arabidopsis* and *Oryza sativa*.

## Results

### Localization of old “prokaryotic” protein encoding genes on chromosomes of arabidopsis and Oryza sativa

**Figure 1:**
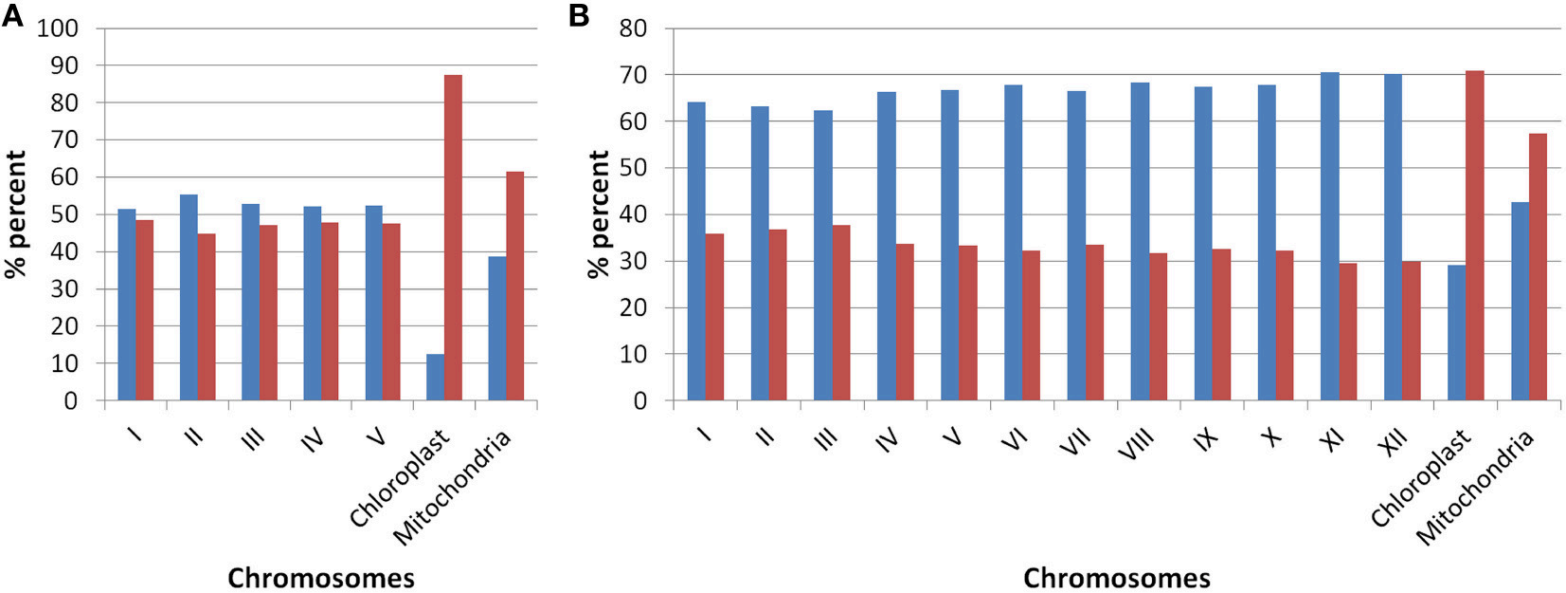
**The plot shows the occurrences of old “prokaryotic” genes on various chromosomes in *Arabidopsis* (A) and *Oryza sativa* (B)**. The bar in the graph depicts the number of genes present on the respective chromosome. The “blue” color shows the total number of new genes while “red” color shows the number of old “prokaryotic” genes present on respective chromosomes.

Our analysis of *Arabidopsis* and *Oryza sativa* genomes with respect to the presence of old “prokaryotic” proteins has revealed several characteristic features of these genomes. Firstly, the fraction of genome which represents the old “prokaryotic” proteins is relatively higher in *Arabidopsis* than in *Oryza sativa* (Figure 1). With respect to total genes present in *Oryza sativa*, 36.5% protein encoding genes were found to be old “prokaryotic” protein encoding genes while in *Arabidopsis*, 49.3% of the total proteins were old “prokaryotic” protein encoding genes (Supplementary Table 1). The percentage occurrence of the old “prokaryotic” protein encoding genes in other chromosome with the respect to the total number of genes present per chromosome ranged from 44 to 48% in *Arabidopsis* with the lowest number of these being present on chromosome II. Further, chromosome I possessed the highest number of such genes, that is, 48%. In *Oryza sativa*, the minimum number of old “prokaryotic” protein encoding genes was observed on chromosome XI and XII (29%) while maximum number of these was observed on chromosome III and II (37 and 36%, respectively). In *Oryza sativa*, chromosome X is considered as the smallest chromosome but its average gene density is comparable to chromosome I and IV (Rice Chromosome 10 Sequencing Consortium, 2003). The proportion of the old “prokaryotic” protein encoding genes on chromosomes X was found to be similar to chromosome IV (33%). In chloroplast, the old “prokaryotic” protein encoding genes were 87.5 and 70.8% of the total genes present in the chloroplast of *Arabidopsis* and *Oryza sativa*, respectively. In mitochondria, the old “prokaryotic” protein encoding genes were 61.4 and 57.4% of the total genes present in the mitochondria of *Arabidopsis* and *Oryza sativa*, respectively. The large fraction of old “prokaryotic” genes in the chloroplast and mitochondria genome of both *Arabidopsis* and *Oryza sativa* supports their endo-symbiotic origin. In general, the old “prokaryotic” protein encoding genes were not concentrated on any specific chromosome in the two genomes.

Analysis of duplications in the genes encoding old “prokaryotic” proteins showed that there were more in *Arabidopsis* (14.98%) than in *Oryza sativa* (7.75%). Similarly, the number of introns present in genes encoding old “prokaryotic” proteins was found to be 67% in *Arabidopsis* in comparison to 51.66% in *Oryza sativa*. However, the number of introns in genes encoding new proteins were found to be greater in *Oryza sativa* (48%) than in *Arabidopsis* (32.9%) which may indicate their role on its genome evolution.

### The multi-domain architecture

**Figure 2:**
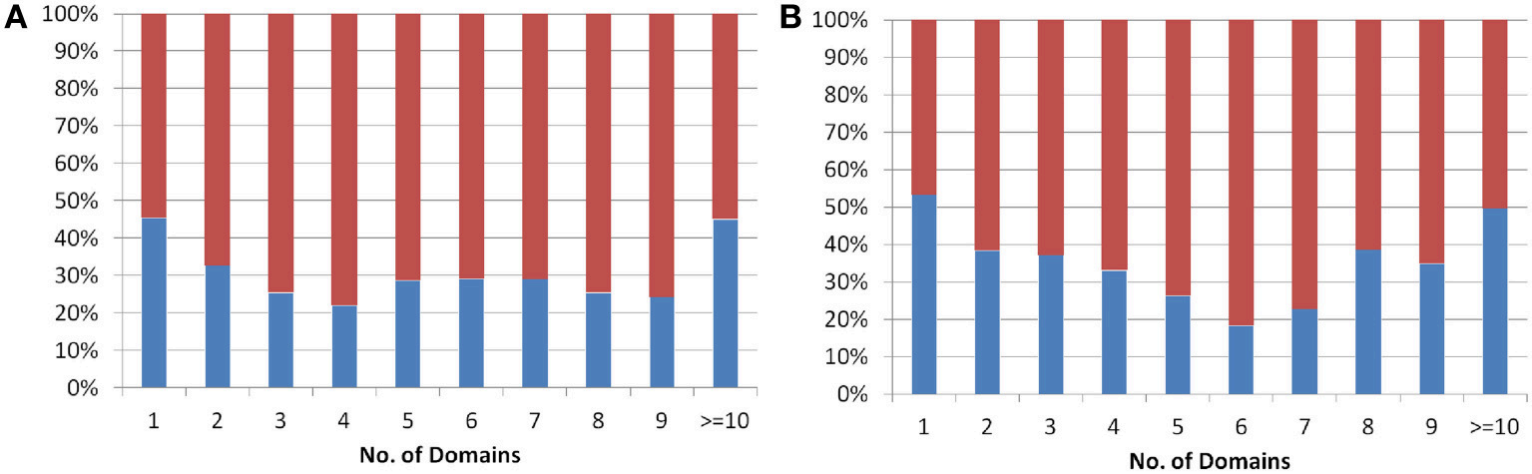
**The plot shows the percentage of proteins having one or more than one domain in the old prokaryotic proteins (red) and in new proteins (blue) in *Arabidopsis* (A) and *Oryza sativa* (B)**.

The protein domains are considered as independent evolutionary units, which either have independent function or play a supportive role in multi-domain architecture (Apic et al., 2001; Vogel et al., 2004) in eukaryotes. The domain architecture for both *Arabidopsis* and *Oryza sativa* proteins was obtained from the genome-wide prediction using Pfam database (See Methods). The analysis showed that single domain old “prokaryotic” proteins constitute ~50% of the total old “prokaryotic” proteins in both *Arabidopsis* and *Oryza sativa*. In order to analyze multi-domain architecture in both *Arabidopsis* and *Oryza sativa*, domain architecture pattern of both new and old proteins was compared (Figure 2). Interestingly, the old “prokaryotic” proteins with 1-domain and >10-domains were found to be equal proportion in both *Arabidopsis* and *Oryza sativa*.

In order to analyze the distribution of domain architecture patterns in *Arabidopsis* and *Oryza sativa* as well as in old and new proteins, we performed two-sample Kolmogorov-Smirnov test (Massey, 1951). The test results failed to reject the null hypothesis (H_0_, with significance level 0.05) which suggests that the domain architecture in proteins both in *Arabidopsis* and *Oryza sativa* have statistically similar distribution (Supplementary Figure 1). This was evident from the cumulative distribution function plot of the protein domain architecture in these genomes. We observed that the results stands true for all the combinations such as old “prokaryotic” proteins (*Arabidopsis* and *Oryza sativa*), new proteins (*Arabidopsis* and *Oryza sativa*), *Oryza sativa* (old “prokaryotic” and new proteins), and *Arabidopsis* (old “prokaryotic” and new proteins). These results suggest that the proteins in *Arabidopsis* and *Oryza sativa* have conserved domain architecture.

### Localization of old “prokaryotic” proteins

**Figure 3:**
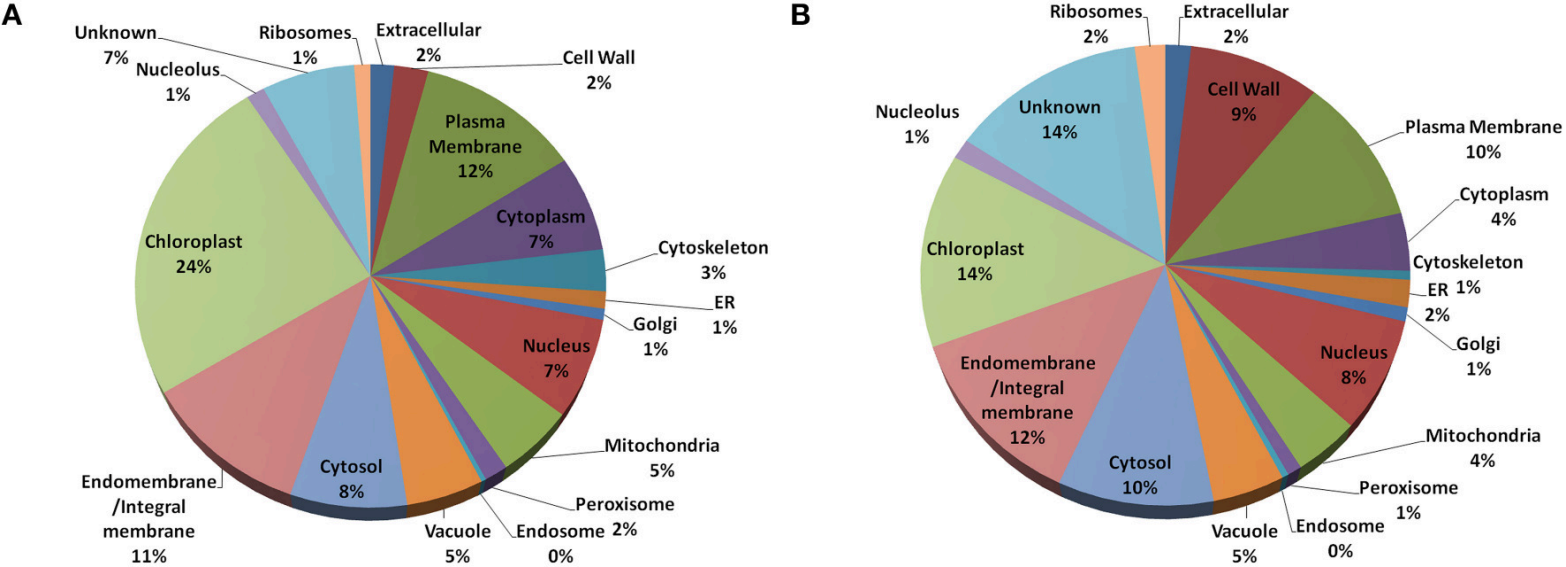
**The pie-chart of the predicted cellular localization obtained using the gene ontology annotation for old “prokaryotic” proteins in *Arabidopsis* (A) and *Oryza sativa* (B)**. In both, *Arabidopsis* and *Oryza sativa*, old prokaryotic proteins were predicted to be localized in the chloroplast and membranous regions.

In *Arabidopsis*, highest fraction (24%) of the old “prokaryotic” proteins were found to be localized in chloroplast followed by plasma membrane and endo/integral membrane (12 and 11%, respectively) while in *Oryza sativa* 14% of the old “prokaryotic” proteins were localized in chloroplast and 12% were localized in endo- and integral membrane (Figure 3). The old “prokaryotic” proteins localized in mitochondria were found to be 4 and 5% in *Arabidopsis* and *Oryza sativa*, respectively. Further, the old “prokaryotic” proteins in nucleus were found to be 8 and 7% in *Arabidopsis* and *Oryza sativa*, respectively. It was observed that more number of the old “prokaryotic” proteins was targeted toward the cell wall in *Oryza sativa* (9%) than in *Arabidopsis* (2%). Localization could not be ascertained for 14% of the old “prokaryotic” proteins in *Oryza sativa* and 7% in *Arabidopsis*. Analysis of the subcellular localization suggests the distinctness of the old “prokaryotic” proteins.

### Functions of old “prokaryotic” proteins

**Figure 4:**
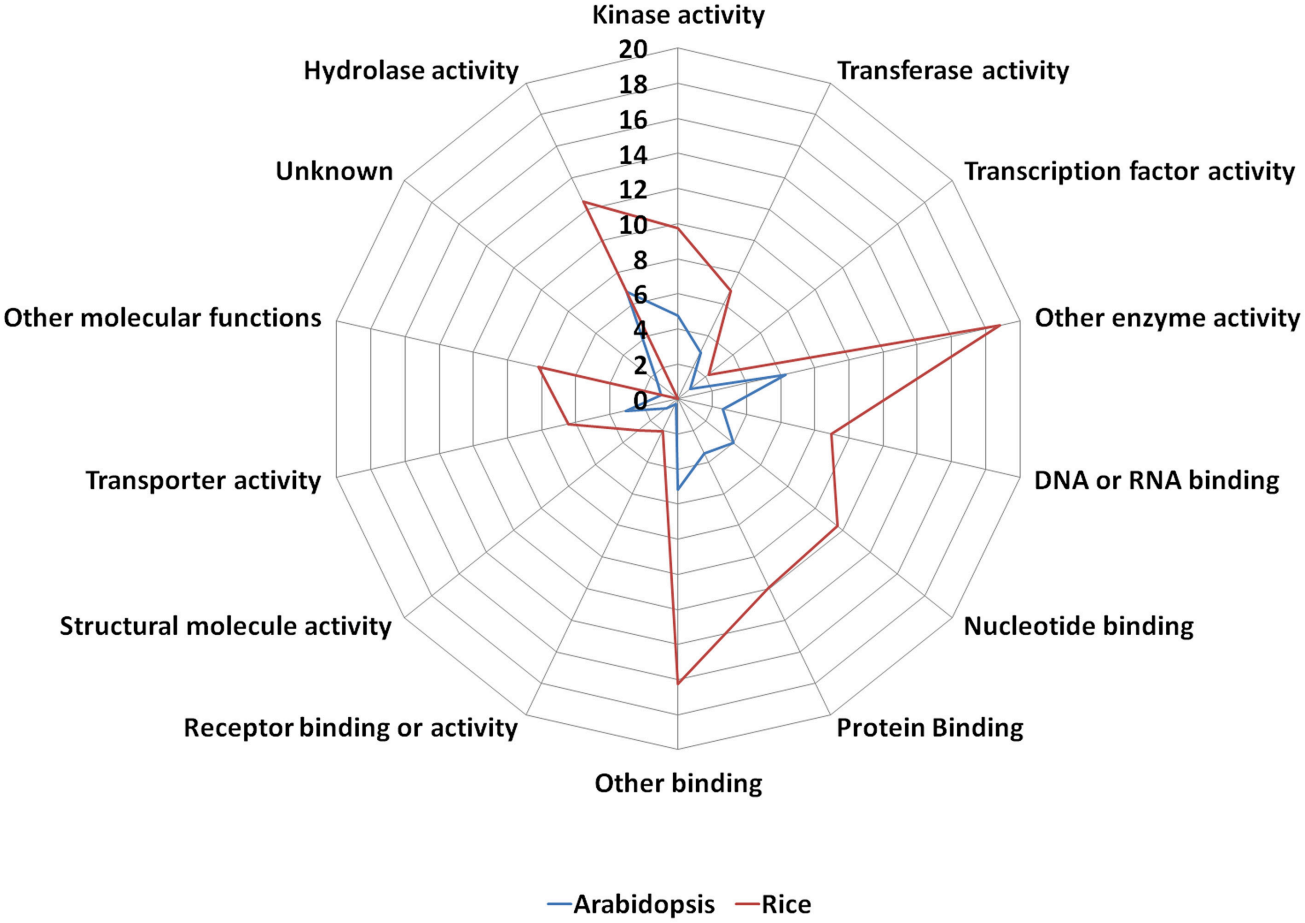
**The function of old “prokaryotic” proteins in *Arabidopsis* (blue) and *Oryza sativa* (red) showing the difference in the magnitude of proteins involved in various activities in the two plant species**. The numbers represent the percentage of old “prokaryotic” proteins involved in various activities in the two plant species. It is interesting to note that the overall trend for the function in the two plant species remains similar. The functional annotations were obtained using the gene ontology annotation for both *Arabidopsis* and *Oryza sativa*.

The old “prokaryotic” proteins were found to be involved in various binding activities such as DNA and RNA binding, protein binding, nucleotide binding etc. in both these genomes (Figure 4). Apart from the binding activity, the old “prokaryotic” proteins were observed to be involved in various enzyme activities related to various metabolic pathways such as oxidation/reduction pathways, regulatory functions in these genomes. Many of these old “prokaryotic” genes were involved in hydrolase and kinase activities in both rice and *Arabidopsis*. Further, these old “prokaryotic” proteins showed similar involvement in various other functions in both the genomes. Those old “prokaryotic” proteins having similar domain architecture in the two genomes but having varied functions has been summarized as Supplementary Table 2.

### Processes involving old “prokaryotic” proteins

**Figure 5:**
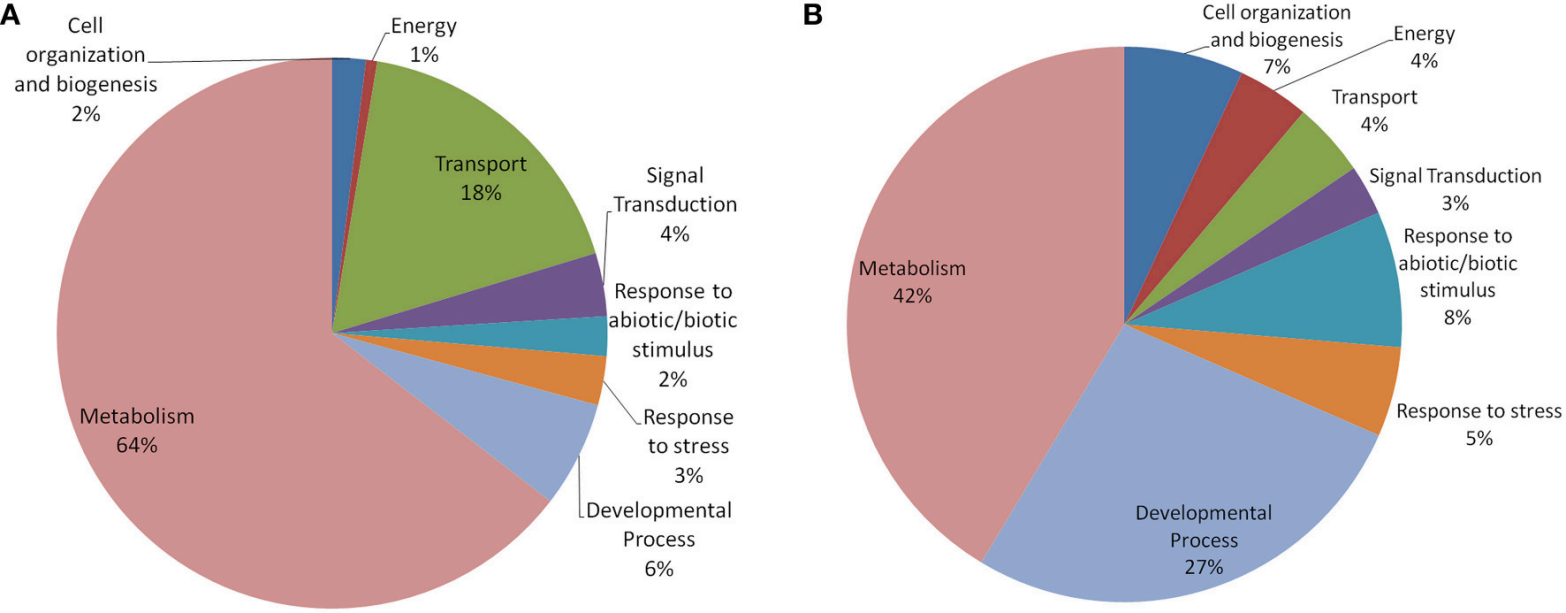
**The pie-chart of the biological processes in which the old “prokaryotic” proteins were found to be involved in *Arabidopsis* (A) and *Oryza sativa* (B)**. The ontologies of the old “prokaryotic” proteins were obtained using the GOSlim assignments for both *Arabidopsis* and *Oryza sativa*. The chart shows the processes in which old “prokaryotic” proteins are involved have got diversified in *Oryza sativa*.

In both *Arabidopsis* and *Oryza sativa*, the old “prokaryotic” proteins were found to be involved primarily in metabolic processes (42% in *Oryza sativa* and 64% in *Arabidopsis*; Figure 5). In *Oryza sativa*, 27% of old “prokaryotic” proteins were involved in developmental process while only 6% proteins in *Arabidopsis* were found in this category. Owing to the greater involvement in the developmental process, more number of old “prokaryotic” proteins in *Oryza sativa* (7%) were involved in cell organization and biogenesis process in comparison to 2% in *Arabidopsis*. Proteins involved in transportation process were found to have prominence in *Arabidopsis* (18%) in comparison to only 4% in *Oryza sativa*. In the stress responsive function (abiotic or biotic), old “prokaryotic” proteins were found to be 5% in *Oryza sativa* while in *Arabidopsis*, only 3% belong to this group. Further, in *Oryza sativa*, 8% of the old “prokaryotic” proteins were involved in response to abiotic or biotic stimulus as compared to 2% in *Arabidopsis*.

### Analysis of family and domains in old “prokaryotic” proteins

**Figure 6:**
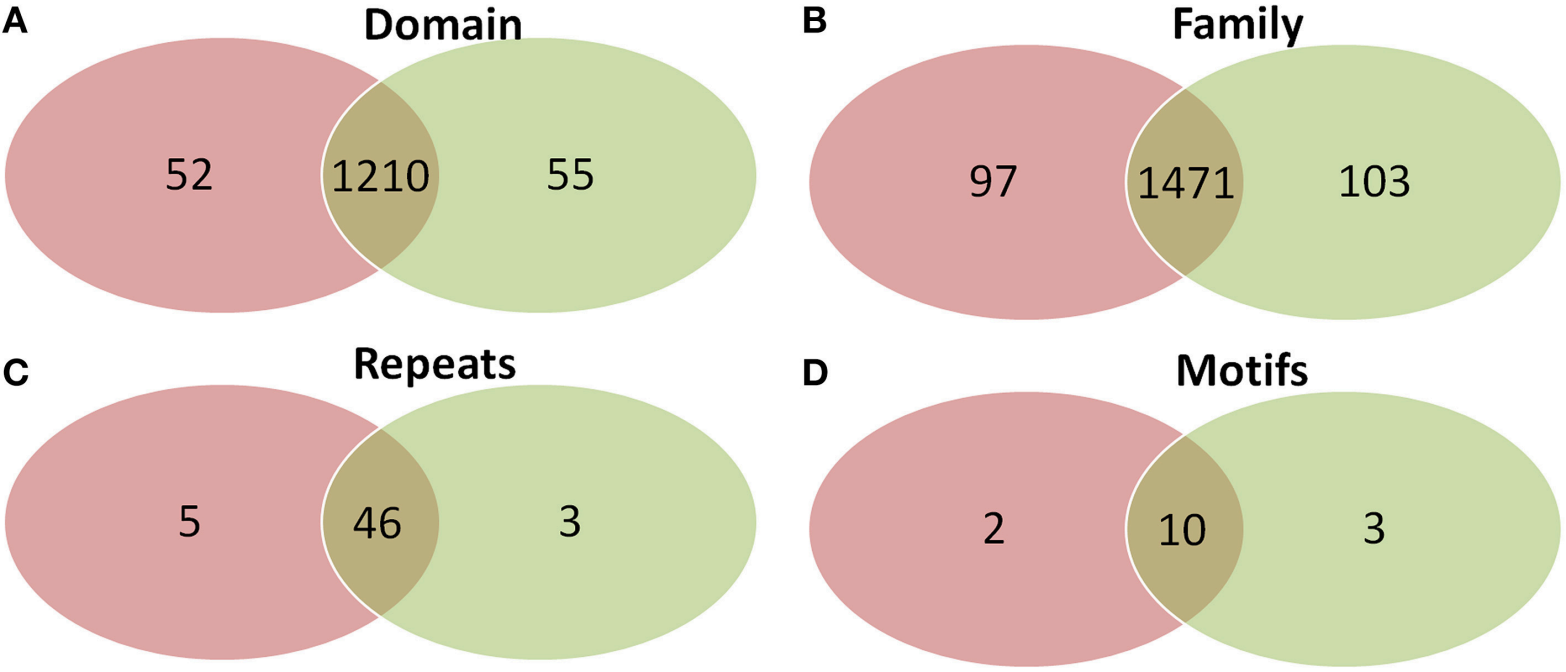
**The Venn diagram of the domains (A), families (B), repeats (C), and motifs (D) present in the in the old “prokaryotic” proteins in *Arabidopsis* (green) and *Oryza sativa* (pink)**. The diagram shows similarity which old “prokaryotic” proteins share with respect to domains, families, repeats, and motifs in the two genomes.

Comparison of domains revealed that old “prokaryotic” proteins of both *Arabidopsis* and *Oryza sativa* share 1210 domains while 52 and 55 domains were found unique to *Arabidopsis* and *Oryza sativa*, respectively (Figure 6). Similar was the case for gene families present among the old “prokaryotic” proteins of both these genomes. The total of 1471 families were observed as common, while 97 families in *Arabidopsis* and 103 families in *Oryza sativa* were found to be unique. Interestingly, the trend continued for the number of repeats and motifs present in the old “prokaryotic” proteins viz. 46 repeats and 10 motifs were found common among the old “prokaryotic” proteins. Contrastingly, in case of repeats, *Arabidopsis* got more number of repeats i.e., five in comparison to the unique repeats present in *Oryza sativa* (three).

## Discussion

One of the biggest opportunities in the post-genome sequencing era is to dig deep into the genomes, in order to gain insights about the possible role of proteins in various complex processes. Among the monocots, *Oryza sativa* is the model plant for analyzing various agronomic traits while in dicots, *Arabidopsis* is the model crop plants due to its notable characteristics. Earlier reports have given interesting comparative overviews of the number of protein candidates of TCS (Pareek et al., 2006), CDCP (Kushwaha et al., 2009), Gly (Mustafiz et al., 2011), Histone chaperones (Tripathi et al., 2015), and NCX (Singh A. K. et al., 2015) family present in the two genera (*Arabidopsis* and *Oryza sativa*) which gives insights into the possible modes of their genome evolution. One of the major questions, which still remains to be answered, is how the evolution of these proteins, *per-se*, has taken place in the two genomes. The present study has been undertaken to look into these genomes to find the probable answer to this question.

Old “prokaryotic” genes containing ancestral components such as functional and/or structural domains of proteins form a significant fraction of plant genomes. Earlier, it was hypothesized that the endo-symbiotic evolution led to the acquisition of numerous genes from the endo-symbiotic organelles, which later evolved into chloroplast and mitochondria (Martin and Herrmann, 1998; Kurland and Andersson, 2000; Martin et al., 2002). A large number of endo-symbiotic genes were displaced later to the host's nucleus. These genes produce several precursor proteins which are imported into the chloroplast (Martin and Herrmann, 1998; May and Soll, 1999; Cline and Dabney-Smith, 2008; Jarvis, 2008). Similar analysis in *Arabidopsis* and *Oryza sativa* has also been strengthened by the fact that the plastid DNA has been found in abundance in the genomes of *Arabidopsis* and *Oryza sativa* (Shahmuradov et al., 2003; Alexeyenko et al., 2006). Our results from the genome-wide identifications of the old “prokaryotic” proteins has confirmed that large number of “prokaryotic” protein encoding genes have not only remained confined to the chloroplast, but also might have got incorporated into the nuclear genomes of both *Arabidopsis* and *Oryza sativa*. Our analysis has shown that, chromosomes XI and XII in *Oryza sativa*, have fewer old “prokaryotic” proteins as compared to other chromosomes. Previous reports have suggested large number of duplications on these chromosomes. These duplications are known to be coupled with the high density of disease resistance gene clusters (Rice Chromosomes 11 12 Sequencing Consortia, 2005). The prominence of new genes in these chromosomes might be due to presence of fast evolving genes. It has been shown earlier that the fast-evolving genes might be misclassified as new due to the inverse relationship between the evolutionary rate and age of the gene (Elhaik et al., 2006). Also, higher number of old “prokaryotic” proteins in *Arabidopsis* than in *Oryza sativa* indicate toward greater number of fast evolving genes in *Oryza sativa* than in *Arabidopsis*.

Domains are considered as the structural, functional and evolutionary unit of proteins which are known to fold independently into the stable core (Jaenicke, 1987). Domain combination and recombination leads to the formation of new proteins and their functions (Yang and Bourne, 2009). Domain based architecture is essentially considered to be conserved in plants, irrespective of the size of the genome which holds true for all types of protein domain architecture (Zhang et al., 2012). Our results from the analysis confirm that the protein domain architecture remains conserved in both *Arabidopsis* and *Oryza sativa* (in both old and new proteins) though there are differences in the number of proteins between the two species. Further, old “prokaryotic” proteins were found to be sharing a large number of domains and families in the two genomes.

Prokaryotes are essentially considered as the organisms which lack well-defined nucleus and other membrane bound organelles. Therefore, large numbers of old “prokaryotic” proteins were predicted to be localized in the endo-symbiotic organelles, specifically chloroplast. Studies have confirmed that the process of “conservative sorting” might have assisted them to adapt to the host system machinery (Celedon and Cline, 2013). Earlier analysis has established that the large number of nucleus-encoded proteins was targeted toward chloroplast (Leister, 2003; Vojta et al., 2004). In both *Arabidopsis* and *Oryza sativa* the old “prokaryotic” proteins were localized in chloroplast, endo-membranes, which shows their probable prokaryotic origin. Further, results show that significant number of old “prokaryotic” proteins were involved in binding functions such as nucleotide binding and DNA, RNA binding which might be pointing toward their role in the regulation of genes and their expression in response to alteration in various environmental conditions. Other binding functions of the identified old “prokaryotic” proteins in *Arabidopsis* and *Oryza sativa* may signify their role in various signal transduction pathways. Our results showed that in *Oryza sativa*, old “prokaryotic” proteins were involved in metabolic and developmental process and abiotic and biotic stress response while in *Arabidopsis* large number of old “prokaryotic” proteins were involved in the metabolic processes. As reported earlier, several wild genotypes of *Oryza sativa* have been largely investing its metabolic machinery toward the vegetative development process (Shimizu and Itoh, 2012). In *Arabidopsis*, large number of old “prokaryotic” proteins were found to be involved in transport mechanism while in *Oryza sativa* such functions might have transformed into other crucial functions.

## Conclusion

Analysis of old “prokaryotic” proteins in *Arabidopsis* and *Oryza sativa* showed these proteins share not only domains and families but also share similar domain architecture pattern in the two genomes. Also, number of old “prokaryotic” proteins was localized in the endo-symbiotic organelles such as chloroplasts in both *Arabidopsis* and *Oryza sativa*. The old “prokaryotic” proteins were found to be localized in various membrane bound regions and are involved in various binding functions such as nucleotide binding and DNA, RNA binding which might be inferred to have role in various process of regulation of gene expression. In *Oryza sativa*, old “prokaryotic” proteins were involved in metabolic and developmental processes while in *Arabidopsis* these proteins were largely found to be involved in metabolic processes. Thus, it shows that in *Oryza sativa* old “prokaryotic” proteins may have specialized themselves due to compulsory selective selection pressure, to the functions needed for its fitness and survival (for being economical crop). These old “prokaryotic” proteins might have acquired new functions in *Oryza sativa* while maintaining similar domain architecture. These preliminary studies of old “prokaryotic” proteins in two model plants showed functional diversification of proteins. Thus, the conclusions derived in this study can be further extended to other set of monocot and dicot crops and non-crop plants in order to highlight the diversification of proteins having similar domain architecture.

## Author contributions

HK, AP, and SP conceived the idea and designed the experiments. AS and MJ performed the analysis. HK wrote the manuscript. SP and AP edited the manuscript. All the authors approved the final manuscript.

### Conflict of interest statement

The authors declare that the research was conducted in the absence of any commercial or financial relationships that could be construed as a potential conflict of interest.

## Supplementary material

Supplementary Figure 1 **The cumulative distribution function (cdf) plot showing distribution of the domain in old “prokaryotic” proteins (*Arabidopsis* and *Oryza sativa*) (A), new proteins (*Arabidopsis* and *Oryza sativa*) (B), *Oryza sativa* (old “prokaryotic” proteins and new proteins) (C), and *Arabidopsis* (old “prokaryotic” proteins and new proteins) (D)**. The two-sample Kolmogorov-Smirnov test for comparison of the distribution of the domain architecture in proteins in both *Arabidopsis* and *Oryza sativa* showed conserved domain architecture in the two genomes.

Supplementary Table 1 **List of old “prokaryotic” proteins identified in *Arabidopsis* and *Oryza sativa***.

Supplementary Table 2 **Common domain arrangement identified in old “prokaryotic” proteins in *Arabidopsis* and *Oryza sativa* along with their respective functions in the two genomes**.
