# Supplementary figures and images for: Analyses of Old “Prokaryotic” Proteins Indicate Functional Diversification in Arabidopsis and Oryza sativa
Source: Front Plant Sci. 2016 Mar 15;7:304. doi: 10.3389/fpls.2016.00304 (PMC4792156; doi:10.3389/fpls.2016.00304)

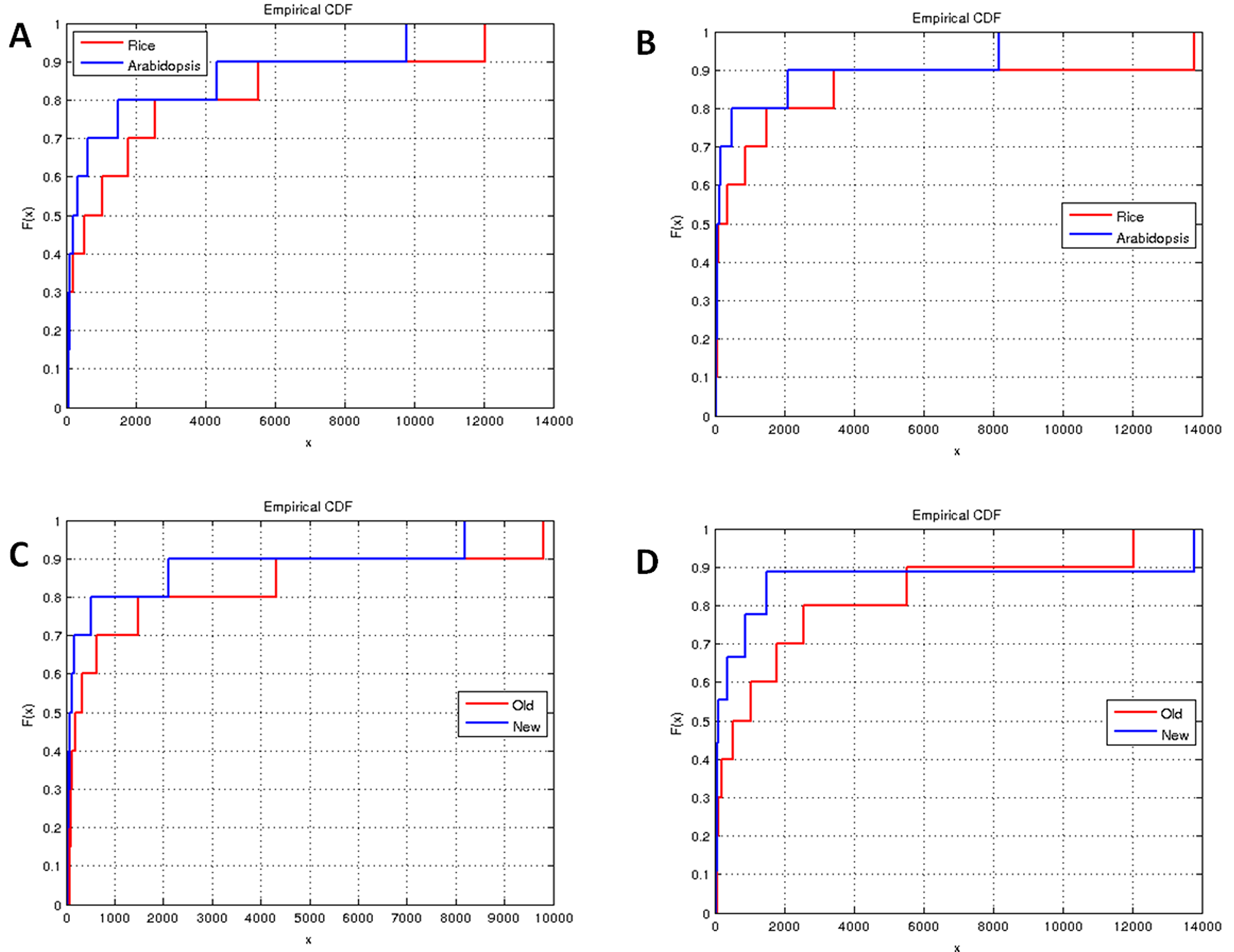

Supplement: Supplementary Figure 1 — The cumulative distribution function (cdf) plot showing distribution of the domain in old “prokaryotic” proteins (Arabidopsis and Oryza sativa) (A), new proteins (Arabidopsis and Oryza sativa) (B), Oryza sativa (old “prokaryotic” proteins and new proteins) (C), and Arabidopsis (old “prokaryotic” proteins and new proteins) (D). The two-sample Kolmogorov-Smirnov test for comparison of the distribution of the domain architecture in proteins in both Arabidopsis and Oryza sativa showed conserved domain architecture in the two genomes. [file Image1.TIF]
